# Effectiveness of Gotu Kola Extract 750 mg and 1000 mg Compared with Folic Acid 3 mg in Improving Vascular Cognitive Impairment after Stroke

**DOI:** 10.1155/2016/2795915

**Published:** 2016-06-01

**Authors:** Kun Marisa Farhana, Rusdy Ghazali Malueka, Samekto Wibowo, Abdul Gofir

**Affiliations:** Department of Neurology, Faculty of Medicine, Universitas Gadjah Mada, Dr. Sardjito General Hospital, Yogyakarta 55284, Indonesia

## Abstract

This study aimed to determine the effectiveness of gotu kola (*Centella asiatica*) in improving cognitive function in patients with vascular cognitive impairment (VCI). This study uses a quasi-experimental design. Subjects in this study were patients with poststroke cognitive impairment who were treated at two hospitals in Yogyakarta, Indonesia. The number of subjects was 48: 17 subjects were treated with 1000 mg/day of gotu kola extract, 17 subjects treated with 750 mg/day of gotu kola extract, and 14 subjects treated with 3 mg/day of folic acid for 6 weeks. A Montreal Cognitive Assessment-Indonesian version (MoCA-Ina) was conducted at the beginning of treatment and after 6 weeks of therapy. It was found that all trials effectively improved poststroke VCI based on MoCA-Ina scores over the course of the study. There is no significant difference in ΔMoCA-Ina (score at the 6th week of treatment − score at the beginning) mean score among the three groups, indicating that gotu kola is as effective as folic acid in improving poststroke VCI. Gotu kola was shown to be more effective than folic acid in improving memory domain. This study suggested that gotu kola extract is effective in improving cognitive function after stroke.

## 1. Introduction

Stroke can cause cognitive decline. The frequency of cognitive impairment after an ischemic stroke ranges from 20 to 30%, with an increasing risk in the two years after stroke [1]. In their research, Ballard et al. found that 25% of patients suffered from poststroke dementia, and the risk of poststroke patients developing dementia within the following five years is nine times higher than in the healthy population, especially for cognitive domains such as memory and attention [2]. Management of cognitive impairment following cerebrovascular disease should be aimed at the prevention of secondary strokes and specific treatment for the improvement of cognitive function. Secondary stroke prevention includes control of risk factors such as blood pressure, cholesterol levels, and hyperhomocysteinemia.

Homocysteine is an independent risk factor for stroke; incidences of stroke due to hyperhomocysteinemia are followed by cerebral microangiopathy and multiple infarction that can reduce cognitive function in various domains [3]. Ingestion of 0.5–5 mg folic acid per day will decrease serum total homocysteine levels by 15–40% within 6 weeks [4]. Use of neuroprotective drugs, antianxiety medicine, hypnosedatives, and antidepressants has side effects and is expensive [5]. Consequently, there is a trend towards the use of natural medicines, especially in the more effective herbal form rather than the active component (isolation of the pure compound) [6]. One medicinal herb commonly used is gotu kola (*Centella asiatica*). The main group of components in gotu kola is the triterpenes including asiaticoside, madecassoside, asiatic acid, and madecassic acid, which have antioxidant, anti-inflammatory, and antiapoptotic properties [7–9]. This may explain why gotu kola has a positive influence on brain plasticity, as well as in increasing the length of dendrites and an enhancement of hippocampal CA3 neuronal dendritic arborization in mice inflicted with neurodegenerative diseases and memory disorders [10].

The above stated reasons provide the basis for a study on the benefits of using gotu kola extract to improve cognitive function in patients with vascular cognitive impairment (VCI) compared with folic acid, which has been commonly used to treat VCI. The measuring instrument used for this study was the Montreal Cognitive Assessment-Indonesian version (MoCA-Ina).

This study aimed to determine the improvement of cognitive function in patients with vascular cognitive impairment utilizing the Montreal Cognitive Assessment-Indonesian version (MoCA-Ina) after an intake of gotu kola (*Centella asiatica*) 750 mg/day and 1000 mg/day and of folic acid 3 mg/day for 6 weeks.

## 2. Methods

This study used a quasi-experimental design. This study received approval from the Medical and Health Research Ethics Committee (MHREC) at the Faculty of Medicine, Universitas Gadjah Mada, and all research subjects provided informed consent. Subjects were ischemic stroke patients, as identified by head CT scans, admitted to Dr. Sardjito General Hospital and Wates Regional Hospital, Yogyakarta. All subjects received standard medical therapy for ischemic stroke according to the guideline and received neuroprotection. No other complementary medicines were allowed. Patients with MoCA-Ina values ≤ 26, age ≥ 18 years, and good liver function were included in this study. Patients with dependencies, those with symptoms of Parkinson's disease, those with severe visual and hearing impairment, and those taking anticoagulants were excluded. Consecutive sampling method was used in this study. Every subject meeting the criteria of inclusion was selected until the required sample size was achieved. Matching was done based on age and MoCA-Ina score in order to avoid bias.

The subjects were divided into three groups, each given a preparation of gotu kola extract (750 mg/day or 1000 mg/day) or folic acid (3 mg/day) for 6 weeks. The researchers provided the gotu kola extract and folic acid tablets to the study participants. The gotu kola extract was prepared from whole herbs in a standardized dry form with a Certificate of Analysis by a GMP certified facility. The extraction solvent used was ethanol 70% and the extract ratio was 10 : 1. The gotu kola extract and folic acid tablets were then encapsulated to obtain the desired dose of 500 mg and 375 mg of gotu kola extract per capsule and 1.5 mg of folic acid per capsule. Each subject consumed 2 × 1 capsules per day. Each subject began treatment following the acute phase of stroke infarction and treatment was completed after 6 weeks. A caregiver was assigned to monitor the patients' compliance with treatment at home. Compliance with the treatment and side effects were monitored by using monitoring forms which were filled out during weekly scheduled visits to the outpatient clinic. Follow-up assessments were also performed by phone for patients who were unable to come to the hospital every week. However, all patients were expected to come to the outpatient clinic after 6 weeks for posttreatment assessment. Laboratory data taken before treatment began included glucose levels, AST, ALT, and INR to determine any contraindications to the proposed treatment.

The measuring tool used to assess the research subjects' cognitive function was the Montreal Cognitive Assessment-Indonesian version (MoCA-Ina). The MoCA-Ina test consists of seven cognitive domains including executive functions, visual spatial abilities, attention and concentration, memory, language, thinking concepts, and orientation. The highest possible score is 30 points, while a score of 26 and above is considered normal. The MoCA test cutoff point is 26; a score equal to or less than 26 signifies cognitive impairment [11]. A MoCA-Ina assessment score was given before the study began and the assessment was carried out again following 6 weeks of treatment.

In this study the participants were not aware of the groups they were in. The MoCA-Ina assessment and other data collection were performed by trained physicians who were blinded to the treatment groups of the patients.

In data analysis, normality test was performed by using the Shapiro-Wilk test. The data that fulfilled the criteria for parametric analysis was analyzed by one-way ANOVA or paired *t*-test, while the data that did not fulfill the criteria for parametric analysis was analyzed by using Wilcoxon signed-rank test or Kruskal-Wallis test. Multivariate analysis was performed to identify effect of confounding variables.

## 3. Results

A total of 51 poststroke patients with cognitive impairment met the inclusion and exclusion criteria involved in this research. There was a loss of follow-up on 3 research subjects: 2 patients did not complete the study and 1 patient discontinued therapy due to an allergic reaction. Forty-eight subjects completed the study: 17 subjects followed a therapy of gotu kola extract 1000 mg/day, 17 subjects followed a therapy of gotu kola extract 750 mg/day, and 14 subjects followed a therapy of folic acid 3 mg/day.

Characteristics of research subject data and MoCA-Ina mean score at baseline are presented in Table 1. There were no significant differences in all variables of the three treatment groups.

The main outcome of this study was to determine the effectiveness of gotu kola extract administered in doses of 750 mg and 1000 mg per day compared to 3 mg of folic acid per day for six weeks for patients with cognitive impairment after stroke infarction. Analysis of MoCA-Ina test results (mean scores) at the end of therapy can be seen in Table 2. All treatment groups showed significant improvement in MoCA-Ina score after six weeks. The mean difference in score of MoCA-Ina at the 6th week minus the baseline for the gotu kola 1000 mg group was 5.6 ± 4.61 (*p* < 0.001; 95% CI), gotu kola 750 mg was 4.94 ± 2.16 (*p* < 0.001; 95% CI), and 3 mg of folic acid was 4.06 ± 3.11 (*p* < 0.001; 95% CI).

Table 3 shows between-group analyses of the effectiveness of treatment on MoCA-Ina scores. It can be seen from the table that the most significant increase in the average score of MoCA-Ina was found in the gotu kola 1000 mg/day treatment group (5.6 ± 4.61). However Kruskal-Wallis analysis showed that not one therapy was statistically more effective than the others in increasing MoCA-Ina score (*p* = 0.39).

A between-group analysis was conducted using Kruskal-Wallis test on the improvements in each cognitive domain on the MoCA-Ina scores. The result showed that there was no significant difference between treatment groups for all domains tested, except for memory domain (delayed recall memory) which showed statistically more significant improvement in patients treated with gotu kola compared with patients treated with folic acid (Table 4).

Secondary outcomes evaluated in this study were the side effects caused by these three types of treatment. According to Pramono and Ajiastuti, common side effects of gotu kola extract therapy are dizziness, headache, abdominal pain, nausea, skin disorders, and drowsiness [12].

Another secondary outcome of therapy that was observed in this study was an increase in the liver enzymes AST and ALT. A paired *t*-test analysis was conducted on AST and ALT levels after 6 weeks of treatment compared to the baseline. The analysis showed no significant difference between AST and ALT levels before and after treatment with *p* value of all groups >0.05 (Table 6).

One-way ANOVA showed no significant difference between the treatment groups on the increase in AST and ALT (Table 7).

Factors that could affect the outcome in this study (i.e., confounding variables) were analyzed by multivariate analysis and given an *R* ^2^ value of 0.25. This value indicates that when combined, confounding variables do not significantly influence the outcome variables as shown in the mean MoCA-Ina scores.  Table 8 illustrates the description of the multivariate analysis of the confounding variables.

Multivariate analysis of confounding variables on ΔMoCA-Ina showed *p* > 0.05 for all variables tested (age, education, gender, duration of DM, and stroke history). This result showed that in this study confounding variables did not affect the outcome of therapy (ΔMoCA-Ina score).

## 4. Discussion

The reliability of MoCA-Ina analysis was previously tested by Hamedan with quite a high kappa value of 0.82 [13]. A breakdown of results for each category is as follows: visual spatial/executive (0.817); naming (0.985); attention (0.969); language (0.990); abstraction (0.957); memory (0.984); and orientation (1.00) [13].

Our study showed that 750 mg and 1000 mg of gotu kola extract or 3 mg of folic acid per day for 6 weeks was effective in improving cognitive impairment after stroke infarction assessed on the increase in MoCA-Ina scores from the beginning to the end of therapy (Table 2). Our between-group analysis did not show any significant difference among treatment groups in terms of MoCA-Ina change. This indicated that gotu kola 1000 mg and 750 mg were equal to folic acid 3 mg in improving cognitive function in vascular cognitive impairment. Previous studies have showed that folic acid supplementation significantly improved cognitive function. Folic acid is thought to work in improving cognitive function through its role in reducing homocysteine level in the blood [14, 15].

Gotu kola has been shown to restore declined cognitive functions in animal models through various mechanisms. The aqueous extract of the entire gotu kola plant has been revealed to show improvement in learning and memory, inhibition of AChE activity, improved dendritic arborization of the amygdala and hippocampus, reduced levels of *β*-amyloid plaques in the hippocampus, decreased oxidative stress, prevention of radiation-induced behavioral changes during clinical radiotherapy, and improved D-galactose induced behavioral, biochemical, and mitochondrial dysfunctions in animals. Asiaticoside, the active constituent of gotu kola, has been reported as an agent to treat dementia and a cognitive enhancer. Asiaticoside has been reported to have therapeutic value against *β*-amyloid neurotoxicity [16].

Studies about gotu kola in humans, however, are still limited. Several studies in humans have showed gotu kola's effectiveness in improving cognitive performance in healthy middle-aged and elderly subjects. In another study gotu kola extract was found to be very effective for improving cognitive impairment in subjects with mild cognitive impairment (MCI) as seen from an improved mean MMSE score of 3.35 with a significance of *p* < 0.01 [17]. The gotu kola was given for 6 months at a dosage of 500 mg twice a day (1000 mg daily). This finding indicates that gotu kola is clinically useful in patients suffering from MCI [16, 18]. The effectiveness of gotu kola extract in improving cognitive functions is related to its cholinergic, antioxidant, and anti-inflammatory properties. Research by Sakina and Dandiya found gotu kola extract to have cholinomimetic activity* in vivo* and also have antioxidant effects. The research also found that the ingestion of gotu kola extract can accelerate the regeneration of nerve cells and increase the elongation of neurites* in vitro* [19].

In this study, after 6 weeks of treatment, a between-group analysis of each cognitive domain showed that there were no significant differences between groups for all domains tested, except for delayed recall memory domain which showed better improvement in patients treated with gotu kola compared with patients treated with folic acid (Table 4). This result showed that gotu kola 750 mg and 1000 mg are equally effective to folic acid 3 mg in improving all cognitive domains in patients with VCI, while being superior in improving memory. In the study of memory function, Sakina and Dandiya found significant improvement in gotu kola extract therapy on day 60 (*p* = 0.01) in both men and women [19, 20]. Cognitive function of delayed recall memory is largely the activity of the prefrontal cortex, the right frontal area, and the biparietal and left cerebellum [21]. Effects of gotu kola extract in the improvement of working memory function, delayed recall memory, and executive function are presumably through modulating the production of dopamine in the prefrontal cortex area, while the long term effects on memory are presumably through modulation of norepinephrine, serotonin, and acetylcholine in the frontal cortex and hippocampus area [22].

Table 5 shows the number and percentage of side effects that emerged during this study. Side effects that arose as a result of a gotu kola extract therapy of 1000 mg/day were constipation and itching (11.11%). The subject who experienced constipation continued with the therapy while the subject who experienced itching discontinued treatment on the 7th day. A side effect that occurred in the gotu kola extract group of 750 mg/day was a bloated feeling (5.5%); that subject continued therapy. The adverse effects of folic acid therapy at 3 mg/day were nausea and heartburn (14.2%). Two subjects who suffered the condition continued the therapy after getting information and symptomatic treatment for their conditions.

Table 6 shows that in all groups no significant differences were found in the levels of AST and ALT after the 6-week treatment period when compared with baseline levels of AST and ALT. Our between-group analysis also did not show difference in AST and ALT changes among the three treatment groups (Table 7). Tiwari et al. also found no significant differences in changes in the levels of AST and ALT after a treatment of gotu kola extract 500 mg twice daily for six months [17]. Meanwhile, O. A. Jorge and A. D. Jorge reported gotu kola hepatotoxicity in 3 patients receiving gotu kola extract therapy for 60 days, but AST and ALT levels returned to normal 2 weeks after discontinuation of gotu kola extract [23].

Multivariate analysis on effect of confounding variables on MoCA-Ina showed *p* > 0.05 for all variables, thus reinforcing evidence that in this study confounding variables did not affect the outcome of therapy. The effectiveness of each therapy was caused by the mechanism of the drug itself. Results of this study are expected to be taken into consideration for clinicians in the use of herbal remedies, especially gotu kola extract, to improve cognitive function in vascular cognitive impairment.

Limitation of this study is that the duration of the follow-up is only six weeks. A longer duration of the study could not only see the effectiveness of therapy but also see the subjects' tolerability of prolonged use of gotu kola extract. Another limitation is that we did not control the patients' diet in the duration of the study.

## 5. Conclusion

This study concluded that a gotu kola extract therapy of 1000 mg/day and 750 mg/day is effective in improving cognitive impairment after stroke infarction but is not more beneficial than a therapy of folic acid 3 mg/day. Among the seven cognitive domains assessed by the MoCA-Ina test, gotu kola treatment showed better improvement in delayed memory recall compared with folic acid treatment. Gotu kola extract either in 750 mg or in 1000 mg was well tolerated with minimal side effects.

## Figures and Tables

**Table 1:** Research subject characteristics.

Variable	All (*n* = 48)	Gotu kola extract therapy 1000 mg (*n* = 17)	Gotu kola extract therapy 750 mg (*n* = 17)	Folic acid therapy 3 mg (*n* = 14)	*p*
Age (mean ± year)	60.27 ± 11.83	60.27 ± 11.91	57.29 ± 10.36	63.13 ± 13.21	0.414

Gender (*n*%)					
(i) Male	60.41	52.94	70.58	57.14	0.481
(ii) Female	39.58	47.05	29.41	42.85	

Education (*n*%)					
(i) Elem	31.3	33.3	23.5	37.5	0.670
(ii) Junior	14.6	26.7	5.9	12.5	
(iii) High	29.2	20.0	52.9	12.5	
(iv) College/univ	25.0	20.0	17.6	37.5	

History of DM					
(i) Yes	37.5	33.3	35.3	43.8	0.823
(ii) No	62.5	66.7	64.7	56.3	

Length of DM					
(i) <5 yrs	53.33	60.0	60.0	40.0	0.804
(ii) >5 yrs	46.67	40.0	40.0	60.0	

History of stroke					
(i) Yes	52.04	58.82	58.82	57.14	0.469
(ii) No	47.91	41.11	41.11	42.85	

MoCA-Ina (mean ± SD)	15.61 ± 7.04	14.60 ± 6.63	16.18 ± 7.51	14.06 ± 8.61	0.188

**Table 2:** Within-group analysis^*∗*^ of the effectiveness of treatment on MoCA-Ina scores.

Parameter	MoCA-Ina at day 0		MoCA-Ina at week 6		ΔMoCA-Ina		*p* ^*∗*^
	Mean	SD	Mean	SD	Mean	SD	
Gotu kola extract therapy 1000 mg/day	14.60	6.66	20.13	5.52	5.6	4.61	<0.001
Gotu kola extract therapy 750 mg/day	16.18	7.51	21.12	6.17	4.94	2.16	<0.001
Folic acid therapy 3 mg/day	14.06	8.69	18.13	8.18	4.06	3.11	<0.001

^*∗*^Wilcoxon signed-rank test was used. ΔMoCA-Ina = MoCA-Ina at week 6 − MoCA-Ina at day 0.

**Table 3:** Between-group analysis^*∗*^ of the effectiveness of treatment on MoCA-Ina scores.

Parameter	Gotu kola extract therapy 1000 mg/day		Gotu kola extract therapy 750 mg/day		Folic acid therapy 3 mg/day		*p* ^*∗*^
	Mean	SD	Mean	SD	Mean	SD	
MoCA-Ina at day 0	14.60	6.66	16.18	7.51	14.06	8.69	0.18
MoCA-Ina at week 6	20.13	5.52	21.12	6.17	18.13	8.18	0.61
ΔMoCA-Ina	5.6	4.61	4.94	2.16	4.06	3.11	0.39

^*∗*^Kruskal-Wallis test was used. ΔMoCA-Ina = MoCA-Ina at week 6 − MoCA-Ina at day 0.

**Table 4:** Between-group analysis^*∗*^ of cognitive domain repair function.

Domain	Gotu kola extract therapy 1000 mg/day		Gotu kola extract therapy 750 mg/day		Folic acid therapy 3 mg/day		*p* ^*∗*^
	Mean	SD	Mean	SD	Mean	SD	
Δexecutive	1.18	1.18	0.88	0.92	0.86	0.86	0.607
Δnaming	0.76	0.90	0.29	0.68	0.21	0.42	0.071
Δ*delayed recall* memory	1.71	1.26	1.59	1.27	0.21	0.42	<0.001
Δattention	0.65	0.99	0.76	0.90	0.57	0.93	0.848
Δlanguage	1.12	0.99	0.59	0.87	0.57	0.93	0.16
Δabstraction	0.12	0.33	0.35	0.49	0.29	0.61	0.354
Δorientation	0.71	1.37	0.71	1.40	0.86	1.26	0.939

^*∗*^Kruskal-Wallis test was used. Δdomain = domain score in week 6 − domain score at day 0.

**Table 5:** Therapy side effects.

Side effect	Gotu kola	Gotu kola	Folic acid
	1000 mg/day	750 mg/day	3 mg/day
	*n* (%)	*n* (%)	*n* (%)
Constipation	1 (5.5%)		
Heartburn			1 (7.1%)
Nausea			1 (7.1%)
Itchiness	1 (5.5%)		
Abdominal bloating		1 (5.5%)	

Total	2 (11.11%)	1 (5.5%)	2 (14.2%)

**Table 6:** Within-group analysis^*∗*^ on the side effects of therapy on levels of AST and ALT.

Parameter	Therapy group	Baseline	After therapy	*p* ^*∗*^
AST	Gotu kola 1000 g/day	23.50 ± 7.75	25.08 ± 5.40	0.55
	Gotu kola 750 mg/day	22.93 ± 7.92	25.86 ± 7.07	0.13
	Folic acid 3 mg/day	20.23 ± 4.45	23.54 ± 4.57	0.08

ALT	Gotu kola 1000 g/day	22.50 ± 9.96	29.58 ± 10.04	0.79
	Gotu kola 750 mg/day	23.71 ± 10.38	24.21 ± 4.59	0.83
	Folic acid 3 mg/day	18.69 ± 6.21	22.23 ± 6.03	0.10

^*∗*^Paired *t*-test was used.

**Table 7:** Between-group analysis^*∗*^ of AST and ALT increases in each of the 3 treatment groups.

Parameter	Gotu kola therapy	Gotu kola therapy	Folic acid therapy	*p*
	1000 mg/day	750 mg/day	3 mg/day	
ΔAST	6.67 ± 13.26	7.06 ± 11.26	3.75 ± 14.13	0.73
ΔALT	11.07 ± 14.05	5.35 ± 16.85	4.36 ± 13.71	0.43

^*∗*^One-way ANOVA was used.

**Table 8:** Multivariate analysis on the effect of confounding variables on the ΔMoCA-Ina outcome.

Variable	Unstandardized coefficients *B*	Standardized coefficients *B*	Sig⁡(*p*)	95% confidence interval	
				Lower limit	Upper limit
Age	−0.060	−0.293	0.40	−0.22	0.099
Education	−0.466	−0.254	0.44	−1.813	0.881
Gender	−1.105	−0.253	0.47	−4.851	2.481
Length of DM	−1.121	−0.254	0.52	−4.976	2.734
History of stroke	−1.679	−0.373	0.34	−5.514	2.156
